# ZFD-Net: Zinc flower defect detection model of galvanized steel surface based on improved YOLOV5

**DOI:** 10.1371/journal.pone.0325507

**Published:** 2025-06-13

**Authors:** Yang Gao, Hanquan Zhang, Lifu Zhu, Feitong Xie, Dong Xiao

**Affiliations:** 1 State Key Laboratory of Digital Steel, Northeastern University, Shenyang, China; 2 College of Information Science and Engineering, Northeastern University, Shenyang, China; 3 Information Technology Center, People’s Hospital of Liaoning Province, Shenyang, China; Manipal Academy of Higher Education, INDIA

## Abstract

Due to the complex factory environment, zinc flower defects and galvanized sheet background are difficult to distinguish, and the production line running speed is fast, the existing detection methods are difficult to meet the needs of real-time detection in terms of accuracy and speed. We propose ZFD-Net, a zinc flower defect detection model on the surface of galvanized sheet based on improved you only look once (YOLO)v5. Firstly, the model combined the YOLOV5 model with our proposed cross stage partial transformer (CSTR) module in this paper to increase the model receptive field and improve the global feature extraction (FE) capability. Secondly, we use bi-directional feature pyramid network (Bi-FPN) weighted bidirectional feature pyramid network to fuse defect details of different levels and scales to improve them. Then we propose a cross resnet simam fasternet (CRSFN) module to improve the reasoning speed of ZFD-Net and ensure the detection effect of zinc flower defects. Finally, we construct a high-quality dataset of zinc flower defect (ZFD) detection on galvanized sheet surface, which solves the problem that no public dataset is available at present. ZFD-Net is compared with state-of-the-art (SOTA) methods on the self-built data set, and its performance indicators are better than all methods.

## 1. Introduction

In the high-end industrial field, any small defect on the surface about galvanized sheet is detrimental to product [1]. However, due to complex characteristics concerning surface for galvanized sheet, research on the detection of ZFDs on its surface is still scarce. Existing research methods are mainly divided into three categories: based on artificial visual detection methods, based on emerging technologies such as infrared and eddy current, and based on deep learning (DL).

The method based on manual visual detection will be subject to the subjective influence of experience and working state of staff, resulting in different evaluation standards and different test results [2]. Due to faster running speed regarding factory production line, and small shape of many steel surface defects, there will be many missing judgments and misjudgments in manual detection, and detection effect is not very ideal. Although emerging technologies such as infrared and eddy current can independently detect a specific defect, they are only suitable for some cases where detection requirements are not high. In addition, these technologies cannot comprehensively assess quality status of products [3]. In complex multi-defect detection work with multiple defects, these techniques may not be adequate [4]. Object detection technology based on DL is an end-to-end detection method, which has been widely used in defect detection in various fields. Many people apply DL to defect detection in steel field.

In recent years, many defect detection methods based on convolution neural network (CNN) have become concerned [5–10]. Liu et al. [11] proposed an improved U-Net model for detection about steel plate surface defects, which solved difficulty about inequality sample collection and segmentation for small defect areas. Zhao et al. [12] based on Faster R-CNN can effectively identify small defects on steel surfaces. Huang et al. [13] proposed SSA-YOLO, which achieves higher accuracy and sensitivity in detecting surface defects about steel strips. Hu et al. [14] extracted key information for texture and geometric shape by analyzing collected defect information concerning continuous casting billet surface, and obtained 83% accuracy by using support vector machine technology. Subsequently, they introduced CNN technology, which has an accuracy of more than 88%, significantly improving effectiveness of defect detection. Zhang et al. [15] designed a detection model named GDM-YOLO, which was derived from improvement of YOLOv8s. The experimental results show that, compared with YOLOv8s, accuracy regarding GADM-YOLO on the Neu-Det dataset is increased by 3%. At the same time, the number of parameters is reduced, and real-time demand for steel surface defect detection in industrial production is reached. Meng et al. [16] proposed an improved method for steel surface detection based on YOLOv8n. By replacing space pyramid fusion module, YOLOv8n improves speed and accuracy of detecting steel surface defects. By using the advanced machine vision system Faster R-CNN, Gu et al. [17] could accurately find various forms of different defects, and could quickly and accurately locate defects according to their different characteristics. To this end, they also introduced pyramid fusion algorithm and deformable convolution technology to greatly improve accuracy concerning Faster R-CNN. The average recognition accuracy is more than 90%, and accuracy is less than 90% when dealing with more complex situations such as scratches and black spots on the edge. Finally, after a study by Xu et al. [18], the YOLO-v3 network model achieved satisfactory results in treating the defects on the surface of the steel plate. MobileNet technology was used to strengthen cavity convolution, which greatly improved detection efficiency about model. Through the introduction for inception architecture, the coverage regarding network is greatly expanded, thus significantly improving accuracy for detection.

In addition, edge detection methods have also been applied in the field of defect identification [19–22]. He et al. [23] improved the Canny algorithm in view of the existing edge detection algorithm’s vulnerability to external interference and fuzzy detection results. In this paper, a new automatic edge detection method for power chip package defect image is proposed, which uses double filter algorithm to remove noise from power chip package defect image and improve image quality. Chen et al. [24] proposed an improved Canny operator to realize the edge detection and target region extraction of defective steel plate images. By introducing bilateral filtering and improved Otsu method, the image denoising and double threshold adaptive adjustment of Canny detector are realized respectively, and the feature index with prior knowledge is constructed to filter the image background and extract the target region. The experimental results show that the proposed method is superior to the traditional edge detection methods in the detection accuracy and robustness of defective steel plate images.

Due to characteristics of fuzzy boundary and more background interference and the shortcomings about existing detection methods, a high-precision real-time detection method for ZFDs on galvanized steel surface, ZFD-Net, was proposed in this paper. This method can not only greatly improve accuracy of detection, but also realize real-time detection. Our significant contributions and originality to this work are summarized below:

(1) We propose a framework for surface defect detection of galvanized sheets called ZFD-Net, which is designed to perform ZFD detection tasks with high accuracy and speed. Since there is no public data set available for this paper, we construct a new dataset for detecting ZFDs on galvanized sheet surfaces. The dataset consists of 2880 real-world images with motion blur, clarity changes, different viewing angles, and dust interference.
(2) We propose that the CSTR module can improve ability about model to obtain global attention, so that model can extract global features for image. Bi-FPN was used for multi-scale feature fusion (MSFF), which effectively fused semantic information and location information for different levels in feature extraction network (FEN).
(3) In this paper, a CRSFN module is proposed to accelerate the model inference speed and improve the model’s ability to focus on important defect features.
(4) Experimental analysis shows that compared with other detection methods, the ZFD-Net algorithm proposed in paper has better performance in detecting ZFDs on surface of galvanized sheet, effectively meeting the requirements of the production line for detection accuracy and detection speed, and has better generalization ability.

## 2. Dataset production and augmentation

### 2.1. Zinc flower defect dataset of galvanized sheet

**Fig 1:**
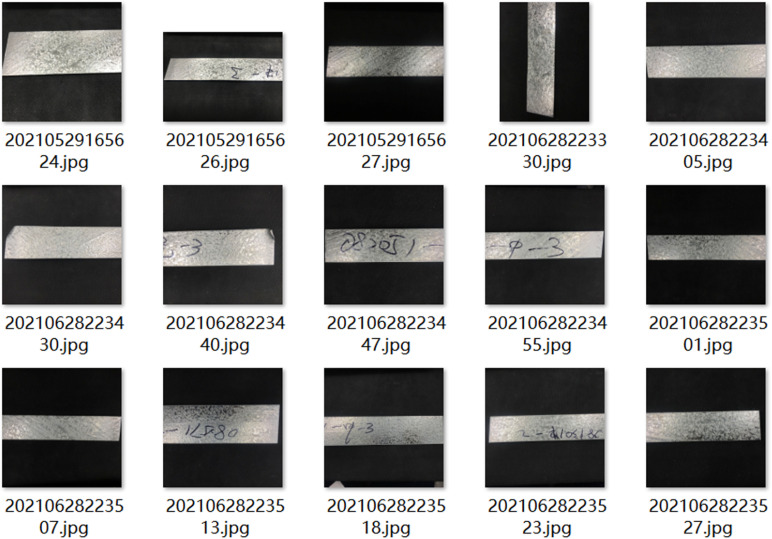
Picture of sample zinc-coated steel.

**Fig 2:**
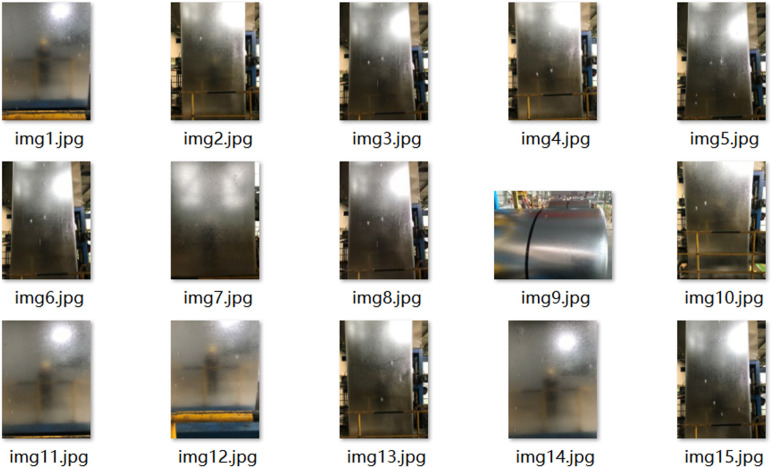
Pictures from the factory production line.

The quality about dataset directly determines performance regarding trained model and upper limit for detection effect. A high-quality dataset is an important prerequisite for verifying performance regarding model. At present, there is no public dataset of ZFDs on surface of galvanized sheet, so data sets used in training and testing for this paper are self-made dataset. In this paper, sample images for defective galvanized sheets and real-time images about intercepted production lines were selected to produce data sets, as shown in Figs 1 and 2.

**Fig 3:**
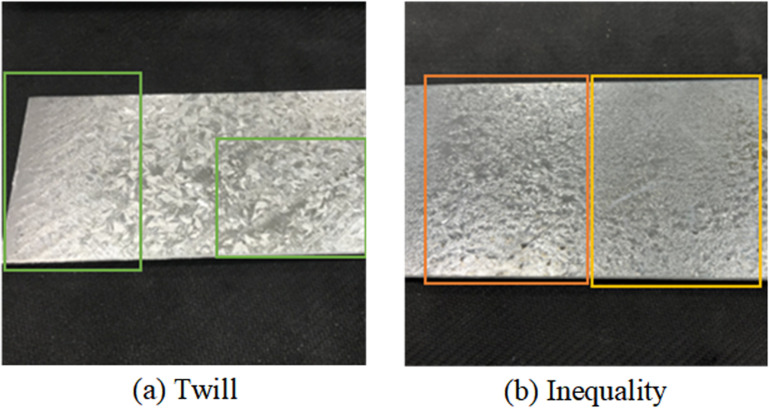
Examples of different defects.

In this paper, the dataset of zinc-flower defects on surface concerning galvanized sheet is collected from the finished products of galvanized sheet produced by the factory. We choose twill and inequality two common defects for research; each picture contains obvious defects. Among them, twill defects mainly occur in hot base galvanized products with thick substrate and thick zinc layer, which are manifested as streaks on edge concerning hot base galvanized sheet at a certain angle to running direction concerning strip steel, as shown in green box about Fig 3a, and zinc layer at streaks is obviously thickened. In extreme cases, streak may run through entire cross section about galvanized sheet, forming a penetrating stripe defect. Inequality zinc flower size is a common defect, it is shown that head and tail concerning steel zinc flower size is significantly smaller than the middle, or difference between the two is large, as shown in yellow and orange boxes in Fig 3b.

Zinc-flower defect data set of galvanized sheets are composed concerning original image and corresponding label files. First, this paper uses Labelimg software to classify defects one by one on original image and manually label them to make labels for different kinds of defects, in which pixels of different defects are marked with different values. In the task of detecting zinc-pattern defects on surface concerning galvanized sheet, twill defects are marked as 0 and inequality defects are marked as 1. txt files suitable for YOLO network format and XML files in VOC2007 format are exported.

### 2.2. Data preprocessing

When galvanized sheet is sampled in factory line, due to high-speed operation concerning equipment, camera used to photograph ZFD on surface about galvanized sheet will move relative to galvanized sheet. In addition, due to problems such as exposure time, motion blur occurs when camera takes a single frame shot. Because image acquisition itself is susceptible to external environment interference, and surface defects of galvanized sheet have characteristics regarding fuzzy boundary, similar to background area material, color, texture, etc., which is easy to cause interference, direct training will lead to poor defect detection effect.

**Fig 4:**
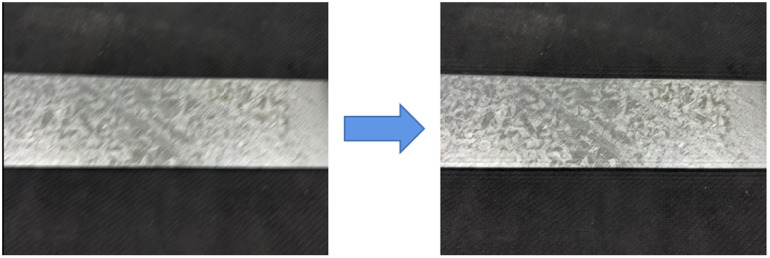
Reduce motion blur.

**Fig 5:**
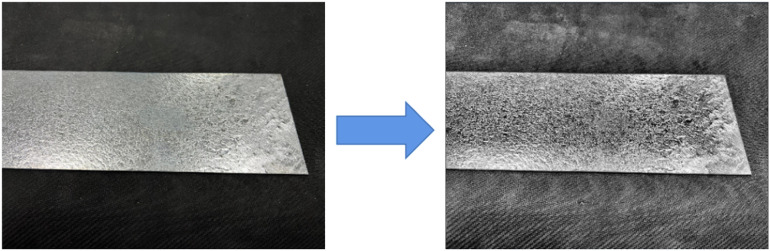
Effect images of CLAHE algorithm.

Therefore, two preprocessing operations, wiener filter [25] to reduce motion blur and contrast limited adaptive histogram equalization (CLAHE) [26], are carried out in this paper according to characteristics regarding galvanized sheet images. It can effectively remove noise and other interfering factors in picture, thus greatly enhancing accuracy for data. Effect concerning reducing motion blur is shown in Fig 4. CLAHE’s shown in Fig 5. As can be seen from Fig 5, after processing with this algorithm, contrast about image is improved, and detailed information such as defect boundary is richer and clearer.

(a) Original motion blur image(b) Remove blur after image

### 2.3. Data enhancement

**Fig 6:**
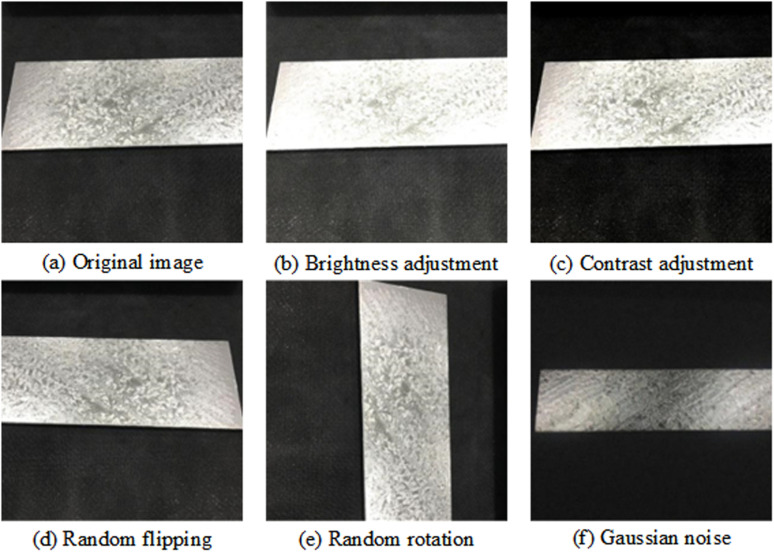
Effect images of data enhancement.

Generally speaking, a large number of data samples are needed to train DL network model so that parameters in network can reach optimal state, and the quality of training samples also greatly affects final performance concerning network. However, in fact, image for ZFD on surface about galvanized sheet can be obtained from production line and defective galvanized sheet, and image is usually too large, resulting in cumbersome data labeling work. Dataset used for training is not enough to support a good detection model, so it is necessary to rely on data enhancement to expand dataset image. To increase diversity of datasets and improve performance concerning model, this paper enhanced marked datasets [27], expanded data sets by random rotation, random flipping, changing brightness, adjusting contrast, adding gaussian noise and other methods, and increased the size concerning dataset to 12 times that about original dataset. It effectively reduces overfitting during training and improves generalization and robustness network. The overall effect is shown in Fig 6. Through the above data enhancement methods, we expanded dataset, and finally obtained 2880 ZFD images and corresponding labels concerning galvanized sheet. Training set, valid set and test set are randomly divided according to proportion of 80%, 10% and 10%. Valid set is used to select optimal combination of network weight parameters. Finally, weight parameter model is applied to test set to evaluate model performance.

## 3. Propose method

### 3.1. The overall architecture of ZFD-Net

**Fig 7:**
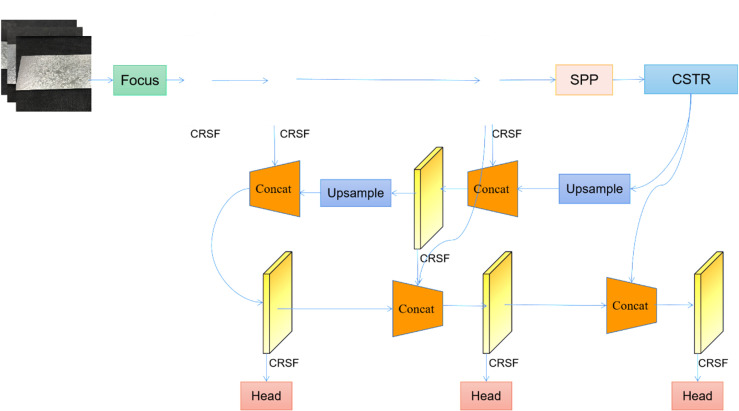
Overall structure of ZFD-Net.

By improving and optimizing the baseline (YOLO-v5), we propose a new ZFD-Net network, whose architecture can be seen from Fig 7, which includes four parts: input, backbone FEN, feature pyramid and detection head. YOLO-v5 extracts images from the incoming network through backbone FEN, fuses image semantic information at the top level with the bottom level using FPN, and enhances the positioning signal at the top level using the bottom-up path enhancement method PANet. However, for the detection task of ZFDs on the surface of galvanized sheet, especially the extraction from high-resolution images, because the ZFDs of galvanized sheet do not have clear edges, many features of the background area of galvanized sheet are similar to those of the defect area, if the shallow layer information is directly added to the high-level features, the extraction results will be very unreliable. Since factory has high requirements for speed concerning production line operation, reasoning speed about YOLO-v5 network should also be further improved.

By replacing C3 module with CRSFN module, the ZFD-Net network proposed in this paper solves problem of too many parameters and too much computation. This method not only improves the inference speed regarding model, but also improves the ability about FE. The CSTR module is added to the last layer for backbone FEN to make model have ability to compare global information regarding feature map and obtain a larger receptive field. Finally, due to the complex zinc pattern path on the surface concerning galvanized sheet, YOLO-v5 cannot interpret deeper features, and cannot clearly extract defect and non-defect areas in the image, and introduce a lot of interference information. By using Bi-FPN technology, we can effectively fuse the information for different scales together, so as to better deal with characteristics concerning zinc flowers on the surface about galvanized sheet, and achieve better transmission and fusion of information.

### 3.2. CSTR module

**Fig 8:**
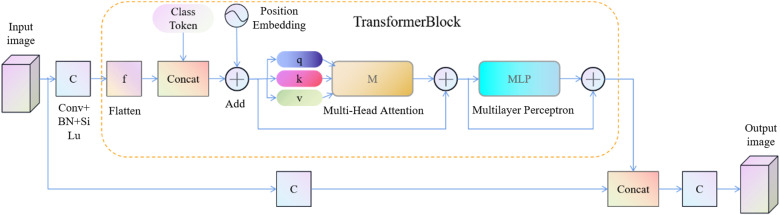
CSTR module structure diagram.

Many characteristics concerning ZFDs on the surface about galvanized sheet are very similar to those of normal zinc flowers, such as similar color, and even similar shape and texture for zinc flowers in inequality defects. It is difficult to accurately identify defects only through the local features concerning feature map. For the task of detecting ZFDs on the surface of galvanized sheet, the network needs a larger receptive field to compare the features regarding different regions, so that the model can learn the features that can truly identify defects. However, the network structure based on CNN is limited by the size concerning convolution kernel. It is difficult to obtain a comprehensive receptive field to extract defect features globally. In addition, the surface of galvanized sheet has the characteristic about reflection, which is affected by light, so it is difficult to identify the exact defect characteristics by common convolutional network. To solve above problems, this paper proposes the CSTR module, which adopts self-attention thought in Transformer, and its global receptive field can extract effective information from the overall feature map. The problem that backgrounds characteristics concerning defects are similar to those of normal galvanized sheet is solved effectively. In this paper, the 1 × 1 convolution method is used to divide the original information into two independent parts, which greatly reduces the computational complexity. One of the branches is stretched into a one-dimensional vector, spliced with the classification vector and then spliced with the position vector. Then encoder module is introduced to extract global features through multi-head self-attention mechanism. Finally, it is spliced with another input branch to form the output of the CSTR module after a convolution process. In this way, the encoder can effectively learn features while reducing module computation and avoiding network degradation. The overall architecture of CSTR module is shown in Fig 8.

### 3.3. Bi-FPN module

**Fig 9:**
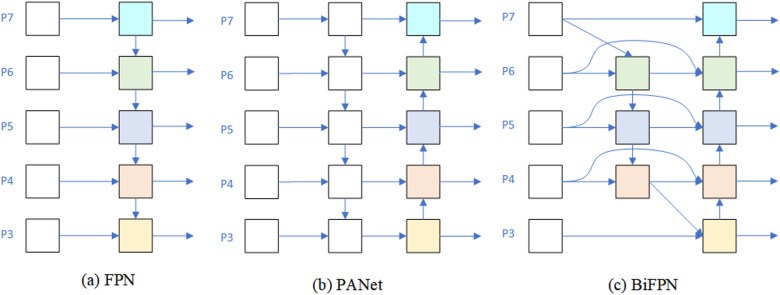
Several features of the pyramid structure.

At present, feature pyramid has become a standard configuration concerning target detection network, which can improve detection ability about objects concerning different scales. Using feature pyramid technology, we can effectively collect feature map information concerning various sizes, which can be effectively integrated into a model, so that we can identify target object more accurately. We used weighted Bi-FPN [28] to do some simple and efficient scale feature fusion, as shown in Fig 9c.

### 3.4. CRSFN module

**Fig 10:**
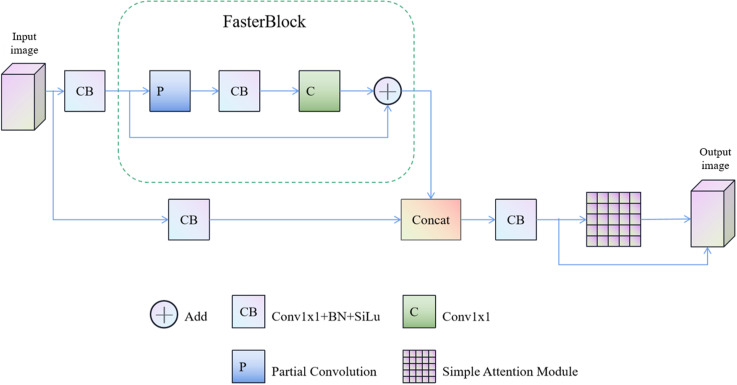
CRSFN module structure diagram.

In the actual industrial production process, the operation speed regarding galvanized sheet production line is very fast, in this case, the operation speed about entire galvanized sheet surface ZFD detection system is also a high requirement. To deal with this problem, we need not only better hardware equipment such as cameras and computers, but also higher reasoning speed of network models. For common convolutional network structures such as Resnet, the reasoning speed regarding model is slow due to large number of parameters and calculation amount, and computing power of the hardware device is also required. In this paper, a new module CRSFN is proposed, which uses a new type of partial convolution (PConv) to reduce the number of parameters and computation. Floating-point operations per second (FLOPS) of the computer is guaranteed, thus speeding up the reasoning speed of the model. In this module, an attention mechanism simple attention module (simAM) based on 3-D weights is used, which can realize cooperative operation on the unified weight for spatial attention and channel attention, and calculate importance about each neuron by optimizing the energy function. Thus, the model can highlight information-rich neurons without increasing the number of parameters, so that CRSFN module can quickly find effective neurons that can express defect characteristics regarding galvanized sheet. Since the influence of attention mechanism on the model may not be applicable to the feature graphs at every level about network, this paper improved simAM and introduced residual structure on its basis, so as to avoid over-interpretation for negative features of the model by simAM. The overall structure of CRSFN module is shown in Fig 10.

In order to make neural networks run faster [29], many lightweight models focus more on reducing floating point operations (FLOPs). In addition, since the number of FLOPs can reflect the computing power of a system, it also becomes an important reference for measuring system performance. Classic lightweight networks such as MobileNets, ShuffleNets, and GhostNet mostly use depthwise convolution (DWConv) or group convolution (GConv). GConv replaces normal convolution to achieve lightweight models. However, these convolution processes are also affected by increase of memory access in the process about reducing the computation amount, resulting in a decrease in FLOPS. However, PConv makes use of the redundancy concerning feature graph. Since the feature graph is highly similar among different channels, conventional convolution is applied to only part regarding input channels and the remaining channels remain unchanged, which can reasonably reduce the number of computation redundancy and memory access. This paper makes use of this characteristic of PConv to accelerate inference speed of the model to cope with the fast running for factory production line.

## 4. Experimental setup

### 4.1. Evaluation criteria

The detection for ZFDs on the surface concerning galvanized sheet can be considered as a classification task, that is, the classification task about defective pixels and other background pixels. In defect detection, using accuracy alone is not an appropriate performance measure. Therefore, in order to accurately evaluate the algorithm, this paper uses Precision, Recall and mean average precision (mAP) to evaluate the results of the detection experiment. Among them, the accuracy rate refers to proportion of ZFDs in the sample predicted by the model, which is actually ZFDs. The recall rate refers to the percentage of samples that are actually ZFDs that are successfully detected by the model. In some cases, accuracy and recall are often contradictory indicators, that is, increasing accuracy tends to lead to lower recall rates, and vice versa. mAP is also often introduced as the main performance indicator to evaluate the accuracy of the target detection model, which is a good compromise between the two significant detection indicators precision and recall. mAP is usually calculated based on Precision-Recall curve (PR Curve). Precision, Recall, AP, and mAP are defined as follows:


$$Precision=\frac{TP}{TP+FP}$$ (1)

$$Recall=\frac{TP}{TP+FN}$$ (2)

$$\begin{array}{c} P_{smooth}(r)=\underset{r^{\prime }>=r}{\max}P(r^{\prime })\\ AP=\frac{1}{11}\sum_{i=0,0.1,...,1.0}P_{smooth}(i) \end{array}$$ (3)

$$mAP=\frac{1}{n_{j}\sum {\mathrm{\backslash nolimits}}_{j=1}^{n_{j}}AP_{j}}$$ (4)

where TP, FP and FN represent true positive, false positive and false negative respectively. For each point on PR curve, value concerning Precision takes value about the largest Precision to right concerning that point. *j* is to the amounts of classes, *n*_*j*_ is total number of classes, and *AP*_*j*_ is average accuracy for each class.

The purpose of each formula used is as follows:

Formula (1) is the Precision, which aims to find out the proportion of actual zinc flower defect samples among all zinc flower defect samples predicted by the model.

Formula (2) is the Recall, which aims to find out the proportion of actual zinc flower defect samples that are successfully predicted correctly by the model.

Formula (3) is the average precision of detecting each defect class, and its purpose is to determine the mean average precision of each defect class.

Formula (4) is the total average precision for detecting all defect classes, and its purpose is to evaluate the total mean average precision of the model for detecting all defect classes.

To meet requirements regarding real-time detection, calculation speed concerning model is also a relatively important index in detection task about ZFDs on surface regarding galvanized sheet. Therefore, reasoning speed regarding model for the whole picture (how many ms is needed to process an image) is also introduced as an evaluation standard in comparison experiment. In this paper, optimal results of each index are shown in bold.

### 4.2. Implementation details

All experiments in this paper were conducted based on the same configuration, as shown in Table 1, to ensure the effectiveness of experimental comparison.

**Table 1:** Experimental environment configuration.

Project	Parameters
CPU	Intel Core i9-10900K @ 3.7 GHz
GPU	NVIDIA RTX 3080
Operating System	Windows 10
Framework	Pytorch 1.9.0
Language	Python 3.8.5
Memory	16GB RAM

**Fig 11:**
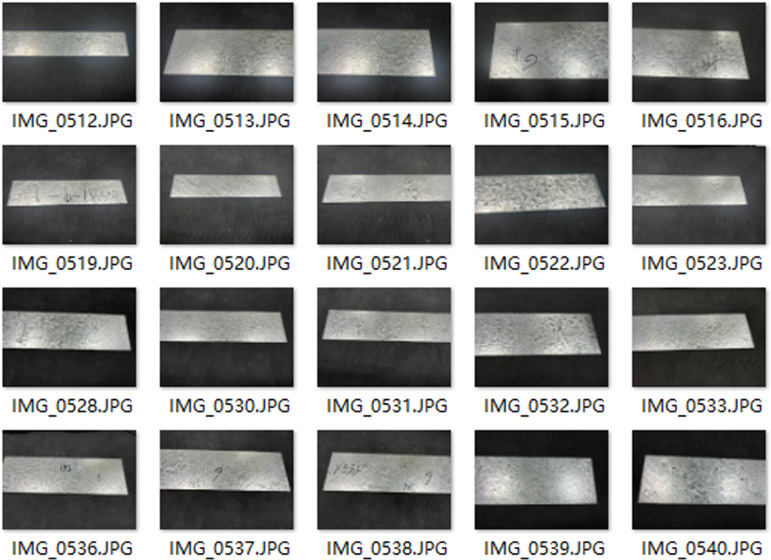
Images of test set.

There are 2880 pictures in our dataset, which are randomly divided into training set, verification set and test set according to proportion regarding 80%, 10% and 10% during the training process. In this paper, 300 epochs iterations were performed on each network participating in experiment, and two main defect twill and inequality data were trained. In this experiment, we first unified the training parameters about model, batch size: 32, momentum: 0.9, image size: 640, weight attenuation: 0.0005, and initial learning rate: 0.01. In this paper, defective galvanized sheet sample images and captured real-time image testset regarding production line were selected to test the performance regarding model. We want to explain that this paper uses 3-channel RGB images for present investigation. Fig 11 is the image taken by the defective galvanized sheet sample about Hangang Group.

## 5. Experimental

### 5.1. Comparison with SOTA methods

We compare the ZFD-Net with other SOTA methods to prove its superiority, and its specific parameters are shown in Table 2.

**Table 2:** Comparison with other mainstream object detection algorithms.

Methods	AP (Twill)	AP (Inequality)	mAP 0.5	mAP 0.5:0.95	Speed	Param	GFLOPs
SSD	0.839	0.934	0.887	0.637	35	3675256	0.695
YOLO-v8	0.941	0.976	0.959	0.814	3.8	9303919	23.2
YOLO-v9	0.971	0.962	0.966	0.855	3.8	6650895	17.4
Efficientdet	0.635	0.789	0.712	0.365	43.6	6101024	4.62
Faster-RCNN	0.783	0.926	0.855	0.555	10	136709509	184.97
YOLO-v10	0.927	0.985	0.956	0.801	5.7	9137726	26.0
ZFD-Net	0.991	0.976	0.983	0.866	3.2	6207647	12.9

The algorithms compared included SSD [30], YOLO-v8, YOLO-v9, Efficientdet, YOLO-v10, and two-stage Faster-RCNN, which were also one-stage target detection networks. Generally, the Efficientdet effect in this dataset was only 0.712 for mAP and 0.887 for SSD algorithm. The detection speed about fax-RCNN was faster than the Efficientdet and SSD, but the number of parameters and FLOPs regarding model were much higher. The overall accuracy for YOLO-v8 and YOLO-v9 is better than SSD, which are 0.959 and 0.966 respectively. The effect regarding YOLO-v10 in the detection task for ZFD on the surface of galvanized sheet is not as good as expected, and mAP is 0.956. Compared with other mainstream algorithms, the algorithm proposed in this paper has improved accuracy concerning twill and inequality defects, and has certain advantages in speed. The results show that this algorithm has obvious advantages in detection concerning ZFDs on the surface about galvanized sheet.

The possible reasons for the decrease in accuracy when YOLO version is added are as follows.

1. Network structure impact: As versions have been increased, YOLO may have introduced new network structures or made adjustments to existing ones. For example, adding more convolutional layers or deeper network structures may cause the model to overfit or be less effective in feature extraction than the previous version, reducing the accuracy of the model.
2. Dataset impact: Dataset splitting, annotation changes, and dataset size all have the potential to affect the model’s ability to generalize as YOLO versions increase, resulting in decreased accuracy as YOLO versions increase.

### 5.2. Ablation study

**Fig 12:**
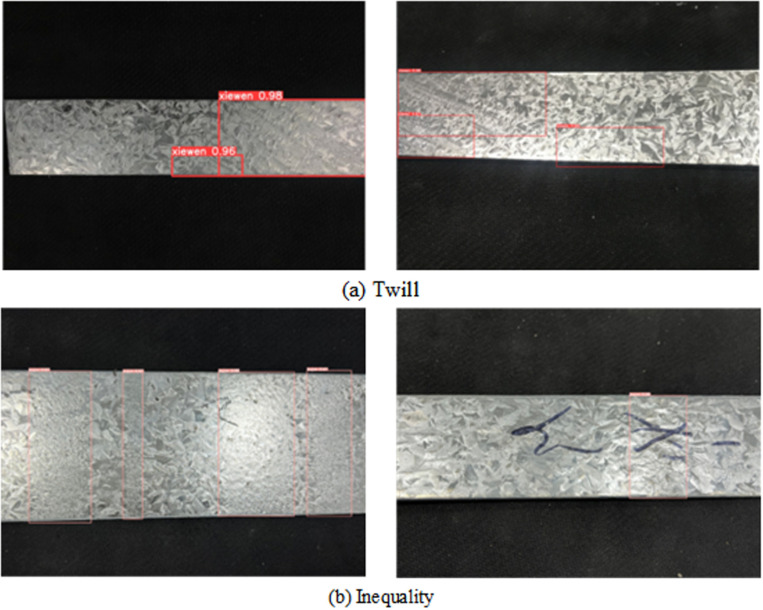
ZFD-Net defects effect.

The ZFD-Net algorithm has achieved excellent results in dataset for ZFDs on the surface of galvanized sheet, and results are shown in Fig 12. There are 2880 pictures in this dataset, including two defect types, twill and inequality, whose accuracy, recall rate and mAP are shown in Table 3.

**Table 3:** Experimental data of ZFD-Net algorithm for defects.

Class	Precision	Recall	mAP 0.5	mAP 0.5:0.95
Twill	0.997	0.925	0.991	0.878
Inequality	0.999	0.924	0.976	0.854
All	0.998	0.924	0.983	0.866

**Fig 13:**
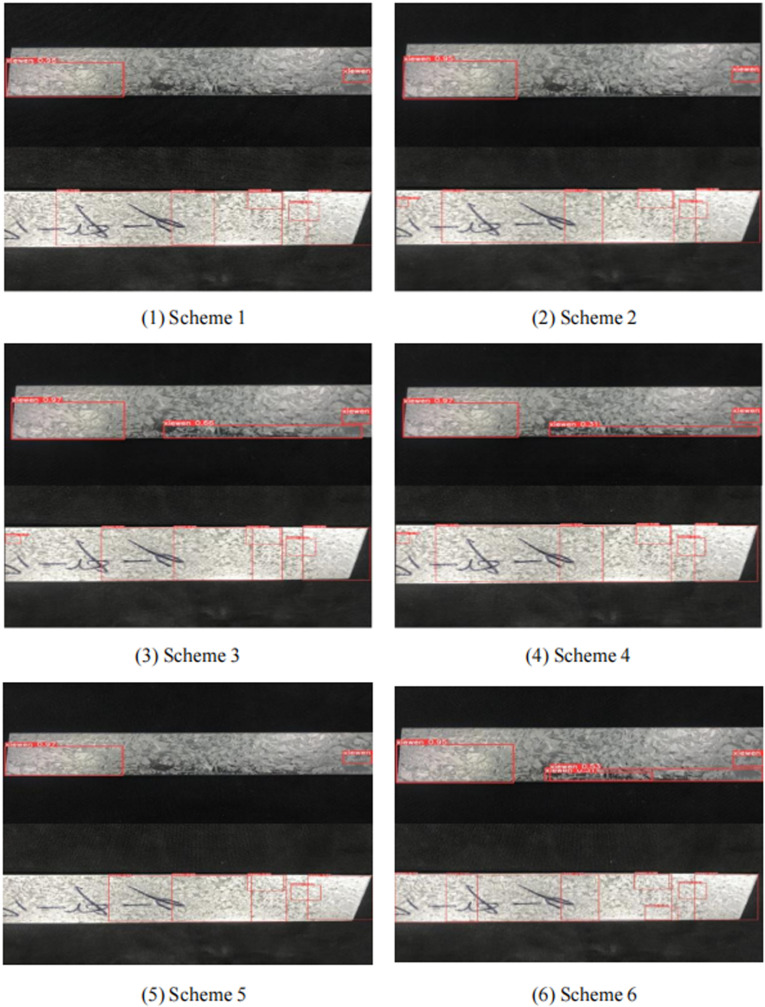
Several schemes to detect twill defects effect.

**Fig 14:**
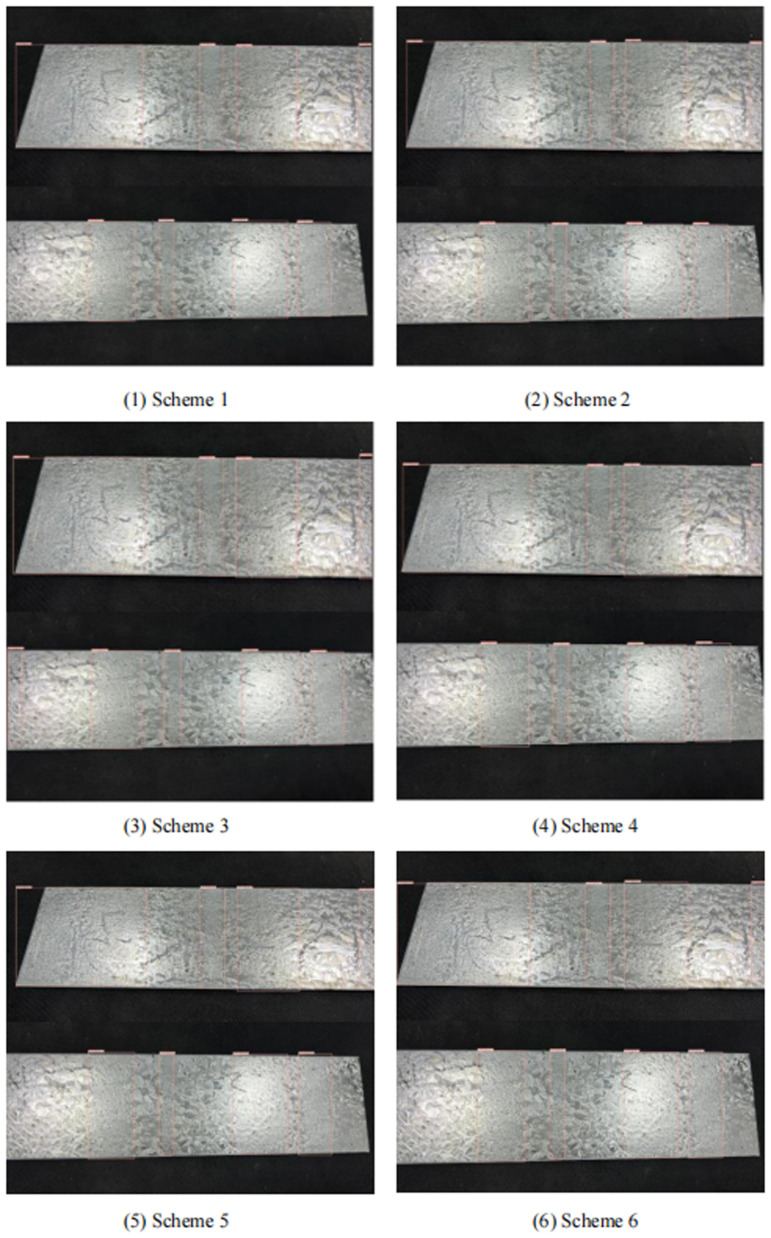
Several schemes to detect inequality defects effect.

As can be seen from Section 3.4, the ZFD-Net surface ZFD detection model proposed in this paper uses CRSFN module to completely replace original position convolutional structure, CSTR module is used in the last layer concerning original YOLO-v5 backbone FEN, and Bi-FPN is used to carry out MSFF about FEN. In this paper, different improvement schemes for YOLO-v5 algorithm are compared on the same dataset, as shown in Table 4. Specific schemes are as follows: (1) Scheme 1 is YOLO-v5. (2) Scheme 2 is YOLO-v5 + CSTR (3) Scheme 3 is Bi-FPN+YOLO-v5. (4) Scheme 4 combines Scheme 2 and Scheme 3 to improve the YOLO-v5 network. (5) Scheme 5 replaces the FEN about YOLO-v5 with a CRSFN module. (6) Scheme 6 synthesizes the previous improvement schemes to generate ZFD-Net model in this paper. The detection results regarding twill defects by six defect detection schemes are shown in Fig 13. The results of six schemes for detecting inequality defects are shown in Fig 14.

**Table 4:** Comparison of experimental results based on different improvement schemes.

Method	Precision	Recall	AP (Twill)	AP (Inequality)	mAP 0.5	mAP 0.5:0.95	Speed	Param	GFLOPs
YOLOv5s	0.972	0.925	0.97	0.948	0.959	0.855	3.4	7056607	16.3
YOLOv5s +CSTR	0.983	0.9	0.984	0.95	0.967	0.855	3.3	7057375	16.1
YOLOv5s +BiFPN	0.935	0.923	0.972	0.969	0.971	0.854	3.3	7122143	16.5
YOLOv5s +CSTR + BiFPN	0.989	**0.934**	0.983	**0.985**	**0.984**	**0.878**	3.3	7122911	16.3
YOLOv5s +CRSFN	0.972	0.93	0.979	0.956	0.968	0.834	**2.3**	**5784991**	**12.6**
YOLOv5s +CSTR + BiFPN +CRSFN	**0.998**	**0.934**	**0.991**	0.976	0.983	0.866	3.2	6207647	12.9

Among them, Scheme 1 May have some missed detection regarding twill defects, and Scheme 2 and Scheme 3 have fewer missed detection cases than Scheme 1, but there are also a few cases in which twill defects cannot be detected. Scheme 4 has a relatively comprehensive detection effect on twill, but detection speed is relatively slow. Scheme 5 uses CRSFN lightweight module, detection speed is fastest among schemes, but there are some cases about missing detection. Scheme 6 used in this paper is best scheme considering detection accuracy and detection speed. For inequality defects, Scheme 3 has some overfitting, and other schemes have similar effects. It can be seen from Scheme 2 in Table 4 that introduction regarding CSTR module into YOLO-v5 model can improve FE capability regarding network, improve AP and average accuracy regarding various categories, reduce the number of FLOPs, and improve calculation speed. It can be seen from data in Scheme 3 that MSFF concerning network using Bi-FPN has a significant improvement on mAP, but FLOPs have increased. Similarly, Scheme 4 has the highest detection accuracy for non-uniform defects. Scheme 5 has the fastest detection speed. Scheme 6 is comprehensive optimal option.

## 6. Conclusion

To solve difficulties caused by unclear edge of ZFD on surface about galvanized sheet and small difference from non-defective background characteristics, a ZFD detection model ZFD-Net on surface of galvanized sheet was established. In this paper, CSTR module is proposed to improve the ability of model to obtain global attention, so that model can extract global features about image. Bi-FPN was used for MSFF, which effectively fused semantic information and location information for different levels in FEN. In this paper, a CRSFN module is proposed to speed up model inference and improve the ability of models to focus on important features. In this paper, CSTR module, Bi-FPN module and CRSFN module are cleverly integrated into YOLO network model. The experimental analysis shows that compared with other target detection networks, ZFD-Net algorithm proposed in this paper has better performance in detecting ZFDs on surface forgalvanized sheet, effectively meets requirements concerning detection accuracy and detection speed regarding production line, and has better generalization ability.
